# Improvement of Pulse Voltage Generated by Wiegand Sensor Through Magnetic-Flux Guidance

**DOI:** 10.3390/s20051408

**Published:** 2020-03-04

**Authors:** Chao Yang, Takafumi Sakai, Tsutomu Yamada, Zenglu Song, Yasushi Takemura

**Affiliations:** 1 Department of Electrical and Computer Engineering, Yokohama National University, Yokohama 240-8501, Japan; 2 School of Electrical Engineering, Nanjing Institute of Industry Technology, Nanjing 210023, China

**Keywords:** Wiegand sensor, large Barkhausen jump, Wiegand pulse, magnetization reversal, Wiegand wire

## Abstract

Magnetization reversal in a Wiegand wire induces a pulse voltage in the pickup coil around the wire, called the Wiegand pulse. The Wiegand sensor features the Wiegand wire and the pickup coil. The amplitude and width of the Wiegand pulse are independent of the frequency of the magnetic-field change. The pulse is generated by the Wiegand sensor, which facilitates the use of the Wiegand sensor as a power supply for equipment without batteries. In order to meet the power consumption requirements, it is necessary to maximize the energy of the pulse signal from the Wiegand sensor, without changing the external field conditions. The distributions of the magnetic field generated from the applied magnet in air and in the Wiegand wire were simulated before the experiments. Simulation predicted an increase in the magnetic flux density through the center of the Wiegand wire. This study determined that the magnetic flux density through the center of the Wiegand wire, the position of the pickup coil, and the angle between the Wiegand sensor and the magnetic induction line were the main factors that affected the energy of a Wiegand pulse. The relationship between these factors and the energy of the Wiegand pulse were obtained.

## 1. Introduction

Recently, the extensive application of wireless monitoring nodes has promoted the effective development and application of the Internet of Things (IoT) [1]. The application fields of the IoT includes industry, agriculture, transportation, medicine, education, and finance [2]. Although wireless nodes use a low-power design, the batteries in the wireless monitoring nodes must be replaced periodically due to their limited battery life. This problem can be solved effectively by using self-generating sensors as power supplies in wireless nodes.

The self-generation of electricity is also called energy harvesting. Sensors collect tiny amounts of energy from their surroundings and they use this as their power source. Presently, the commonly used sensors that are capable of spontaneous electricity generation are the Hall sensor, MR sensor, electromagnetic sensor, and the Wiegand sensor based on the Barkhausen effect [3]. Because the Wiegand sensor does not require an external power supply, it can output sharp and perfect voltage pulses by changing only the polarity of the applied external magnetic field. Moreover, the width of the pulse does not depend on the ratio of the change of the applied external magnetic field [4,5,6]. This feature makes the Wiegand sensor widely popular.

The motivation for this work is that the energy of the pulse signal from the Wiegand sensor can meet the power supply requirements of the wireless nodes, thereby eliminating the need for battery operation. The advantage of using the Wiegand pulse for energy harvesting is that the electrical power is generated by the movement or vibration of a magnet with an extremely slow speed, almost zero [3], which may not be achieved by other vibration-type energy harvesters. A single Wiegand pulse generates an electrical power of 600 nJ [3]. This study attempted to set the ferrite beads for both ends of the Wiegand wire, change the position of the pickup coil around the Wiegand wire, and adjust the direction of the Wiegand sensor in the magnetic field. The magnetic flux through the center of the Wiegand wire increased after the ferrite beads were set at both ends of the Wiegand wire. The simulation results predicted an increase in the magnetic flux density, and it could be estimated that the energy of the Wiegand pulse increased in a corresponding manner. When the pickup coil was closer to the center of the Wiegand wire, the angle between the Wiegand sensor and the magnetic induction line was smaller, and the energy of the Wiegand pulse was larger. Thus, the factors that had a great influence on the energy of the Wiegand pulse were successfully determined. An appropriate configuration was chosen to maximize the energy of the Wiegand pulse without changing the external field conditions.

## 2. Wiegand Sensor

### 2.1. Wiegand Effect

**Figure 1:**
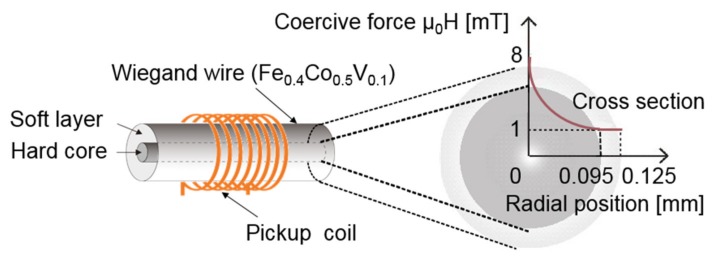
Structure of the Wiegand wire and sensor.

The Wiegand effect was initially observed in magnetic wires of NiFe alloys. In addition, the Wiegand effect was also observed in the magnetic bistable FeSiB amorphous wires. But vicalloy, with a typical composition of Fe_0.4_Co_0.5_V_0.1_, has been known as the optimum material that yields this effect [3]. The diameter of the wire is typically 0.25 mm. Torsion stress, annealing, and cold-treatment were applied to the wire in the fabrication. The strength, twisting and anti-twisting abilities of the Wiegand wire are improved by performing a cold-treatment process. The wire exhibited different magnetic properties near the surface regions and around their center after twisting. This results in a "soft layer" with a soft magnetic appearance and low coercivity of approximately μ_0_H = 1 mT. This also resulted in a hard center, known as the "hard core", with a larger coercive force of approximately from μ_0_H = 1 to 8 mT [7,8,9,10,11,12]. The magnetic properties of the twisted FeCoV wires depend on the conditions of the annealing and torsion stress, which have been previously reported [6]. The volume ratio of the soft layer exhibiting the fast magnetization reversal is calculated to be 23% at a maximum, and the thickness of the soft layer is approximately 0.03 mm, which has been previously reported in detail [3]. The Wiegand sensor consists of two parts: the Wiegand wire, which is the sensitive element of the sensor, and the pickup coil around the Wiegand wire. The structure of the Wiegand wire and the sensor are shown in Figure 1.

**Figure 2:**
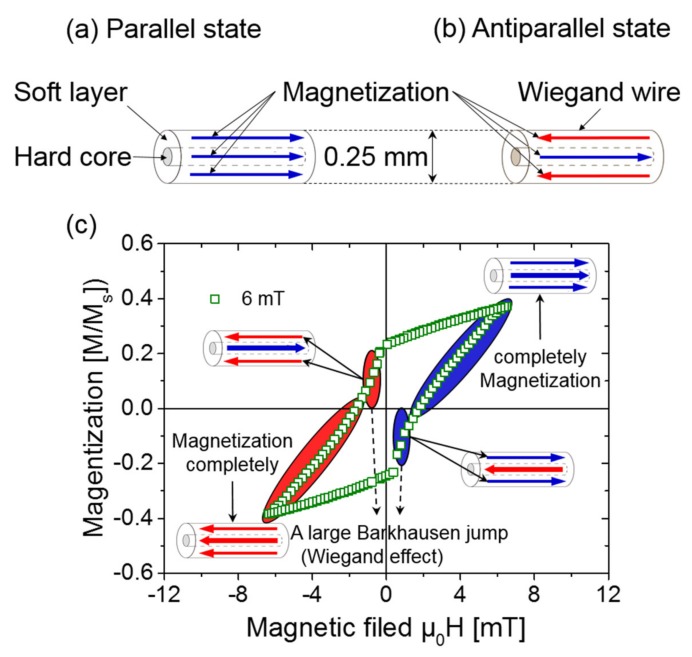
Magnetization states and magnetization curves of the Wiegand wire: (**a**) parallel state; (**b**) antiparallel state and (**c**) magnetization curve (minor loop) of the whole wire.

When no external magnetic field is applied, the hard and soft layers are parallel [13], as shown in Figure 2a. When opposite magnetic fields with the appropriate strengths are applied to the Wiegand wire, the soft layer with the low coercivity will first flip [14,15,16], as demonstrated in Figure 2b. This magnetization reversal of the soft layer is accompanied by a large Barkhausen jump, which is also known as the Wiegand effect [9,10,11]. A magnetization reversal of the soft layer from the antiparallel to the parallel process, against the hard core was observed by applying an asymmetric field, which has been previously reported [3]. As the reverse magnetic field continues to increase, the hard layer with the higher coercivity also reverses subsequently. When the polarity of the external magnetic field changes again, the soft layer becomes reversed, followed by the hard layer in a cyclic process [17,18]. The major loop of the FeCoV wire that is 0.25 mm in diameter and 25 mm in length was measured before the measured minor loop. The saturation magnetization of the sample was M_s_ = 1.78 T. The major loop and minor loop were measured using a vibration sample magnetometer. Figure 2c illustrates the magnetization curve (minor loop) of the whole wire and a schematic of the magnetization process of the Wiegand wire (FeCoV wire) used in this study. It can be seen from Figure 2c that the magnetization reversal is gradual in the hysteresis loop (magnetization curve) measured with an applied static magnetic field up to μ_0_H = 6 mT. A steep change in the magnetization is observed at around μ_0_H = 1 mT, where the magnetization of the soft later is reversed. The magnetization of the hard core is gradually reversed from 1 to 6 mT. The hard core is magnetized completely until the wire reaches the saturation state, which occurs when the applied alternating magnetic field μ_0_H is greater than 15 mT.

### 2.2. Wiegand Pulse and Its Energy

**Figure 3:**
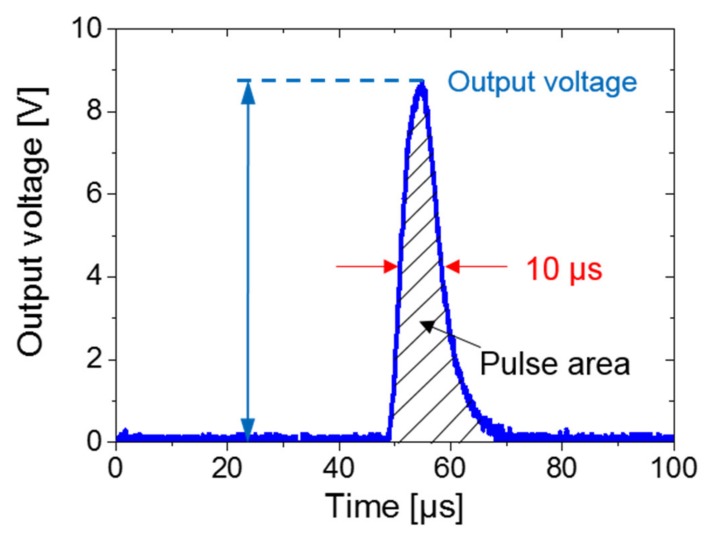
Pulse signal waveform from the Wiegand sensor.

During the magnetization reversal of the soft layer in the FeCoV wire, a voltage pulse that is several volts wide and has a duration of 10 μs was detected in the pickup coil [19,20]. This is depicted in Figure 3, which is called the Wiegand pulse. The pickup coil is 5 mm long and it has 3 000 turns. The external excitation magnetic field is generated by a magnet (NdFeB) with a diameter of 4 mm and a length of 6 mm. The magnetization direction of the magnet used in this study is along the axis.

The energy of the signal can be expressed as:

(1) $$E=\int_{-\infty }^{+\infty }{\left|V(t)\right|}^{2}d_{t}$$

In this study, the energy of the Wiegand pulse is calculated based on the discrete data points determined by the oscilloscope. It can be seen from Equation (1) that the energy of the Wiegand pulse is equal to the sum of the product of the squares of the magnitudes for all discrete points and the time axis. In other words, the energy of the Wiegand pulse is proportional to the area of the shaded part in Figure 3. Because the width of the Wiegand pulse does not depend on the intensity of the applied magnetic field and the frequency of the magnetic-field change, the larger the voltage, the larger the pulse area and the energy of the Wiegand pulse.

## 3. Methods

A Wiegand wire of length 11 mm and a diameter of 0.25 mm, supplied from SWFE, Co. Ltd., Meishan, China, was used in this work. When the Wiegand wire is placed in an alternating magnetic field, the pickup coil repeatedly outputs the alternating Wiegand pulses. Typically, the rotation of a pair of magnets or a single magnet is used to apply an alternating magnetic field to the wire. In this study, a pair of magnets was used. All of the waveforms of the pulse voltage were measured as an open circuit voltage across the pickup coil of the Wiegand sensor.

### 3.1. Enhance the Magnetic Flux

**Figure 4:**
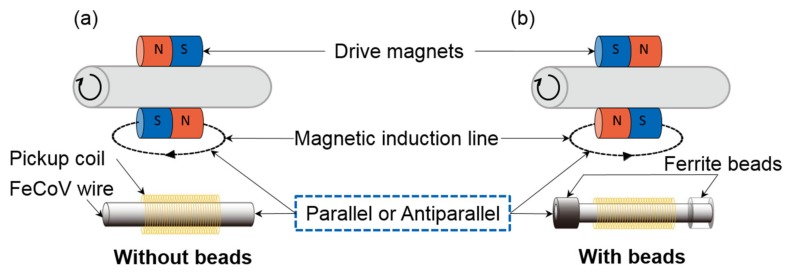
Experimental models: (**a**) “Without beads” model and (**b**) “With beads” model.

Air’s ability to collect the magnetic flux is weak since the magnetic path diverges in air. In contrast, ferrite material is more capable of collecting the magnetic flux in comparison to air. Therefore, in the experiments to enhance the magnetic flux at the center of the Wiegand wire, a ferrite bead was set at both ends of the Wiegand wire as presented in Figure 4. Figure 4a shows a model without ferrite beads, which is referred to as the “Without beads” model. Figure 4b shows the model with ferrite beads, which is referred to as the “With beads” model. The inner and outer diameters of the ferrite beads (TDK, Co. Ltd., Japan, HF57BB3.5X3X1.3) used in the study were 1.3 and 3.5 mm, respectively, the length was 3 mm, and their main metallic elements were Ni, Zn, and Fe.

To study the effects of the magnetic flux density through the center of the Wiegand wire with respect to the energy of the Wiegand pulse, the applied external magnetic-field intensity was set between 4 and 8 mT for both models. Meanwhile, to prove that the ferrite material was more capable of collecting the magnetic flux density, than air, the two models used the same pickup coil and the same external magnetic field. The experimental results of the two models were compared for each additional 1 mT. The pickup coil was wound 200 turns around the Wiegand wire and its length was 5 mm.

### 3.2. Change the Position of the Pickup Coil

**Figure 5:**
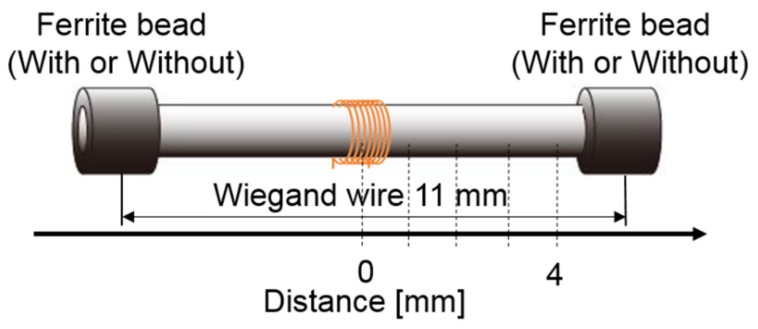
Schematic for position measurement.

To analyze the relationship between the position of the pickup coil and the energy of the Wiegand pulse, a 1 mm long pickup coil was wound 50 times around the Wiegand wire, and an external magnetic field with an intensity of 4 mT was applied. In the initial state, the pickup coil was located at the center of the Wiegand wire and it was moved by a 1 mm step to the right for both models. The pulse waveforms induced by the pickup coil at different positions were measured for the different models. The schematic for the position measurement is shown in Figure 5.

### 3.3. Adjust the Angle of the Sensor in the Magnetic Field

**Figure 6:**
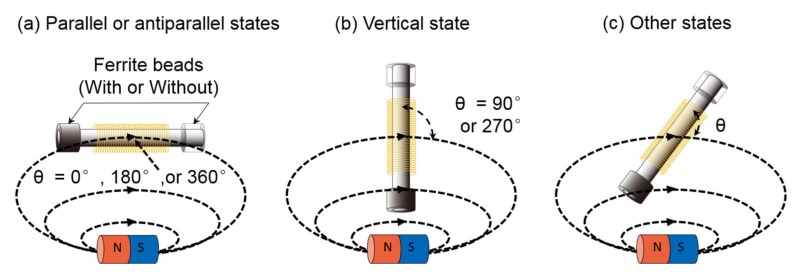
Angle (theta) diagram of the Wiegand sensor in the magnetic field: (**a**) parallel or antiparallel states, (**b**) vertical state, and (**c**) other states.

In order to obtain the relationship between the angle of the Wiegand sensor and the magnetic induction line, as well as the energy of the Wiegand pulse, the waveforms of the Wiegand pulse were detected by the pickup coil. These were measured under the rising and falling triggering modes of the oscilloscope. The angles (between the Wiegand sensor and the magnetic induction line) were 0°(360°), 30°(330°), 45°(315°), 60°(300°), 90°(270°), 120°(240°), 135°(225°), 150°(210°), and 180°, for both models (“With beads” and “Without beads”). The angle (theta) diagram of the Wiegand sensor in the magnetic field is illustrated in Figure 6. The waveforms were then analyzed and compared. The pickup coil was wound 500 times around the Wiegand wire, and its length was 5 mm. The distance between the magnet surface and the center of the pickup coil was 5 mm.

### 3.4. Simulate the Distribution of the Magnetic Field

**Figure 7:**
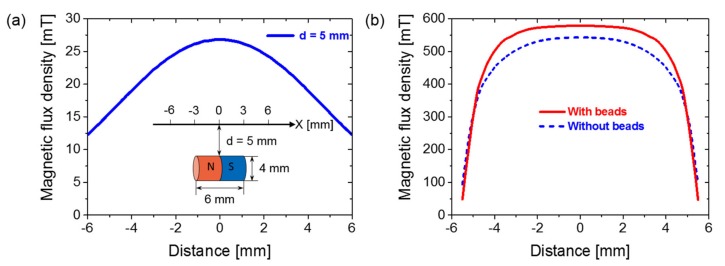
Simulation results of the magnetic flux density in the air and the Wiegand wire: (**a**) in the air and (**b**) in the Wiegand wire.

Before the experiments, the magnetic flux density through the center of the Wiegand wire was simulated by the finite element analysis using ANSYS Maxwell^®^ (ANSYS, Inc., USA). In the simulation, the 3D models of the magnet, the Wiegand wire, the ferrite beads and the air field were defined. The distance from the surface of the magnet to the center of the Wiegand wire was 5 mm. The dimensions of the magnet, the Wiegand wire and the ferrite beads were designed according to the samples used in the experiments, and the air field was designed as a cylinder with a radius of 15 mm and a height of 20 mm. The maximum length of elements of the Wiegand wire, the ferrite beads, the magnet and the air field were set to 0.3, 0.3, 0.5 and 1 mm in the mesh operations, respectively. The corresponding materials were determined according to the samples, as follows: “NdFe35” was chosen for the magnet from the material database of the software, and default parameters were used. A material named FeCoV was defined for the Wiegand wire. Its relative permeability and saturation magnetic flux density were 20 and 1.8 T, respectively, which were provided by the supplier (SWFE, Co. Ltd., Meishan, China). The “ferrite” material in the material database was selected for the ferrite beads, and the parameters were modified according to the sample datasheet of the supplier (TDK, Co. Ltd., Japan). The simulation results in Figure 7a show the magnetic field distribution at a distance of 5 mm from the surface of the magnet that was used in this study. The simulation results in Figure 7b show the magnetic flux density through the center of the Wiegand wire in the “With beads” model and “Without beads” model. The results of Figure 7b verified the judgment that the magnetic flux density through the center of the Wiegand wire was increased by setting a ferrite bead at both ends of the Wiegand wire. This was achieved without changing the intensity of the external magnetic field. This demonstrates the feasibility and effectiveness of the experimental method used in this study.

## 4. Results and Discussion

### 4.1. Relationship between Magnetic Flux and Energy

**Figure 8:**
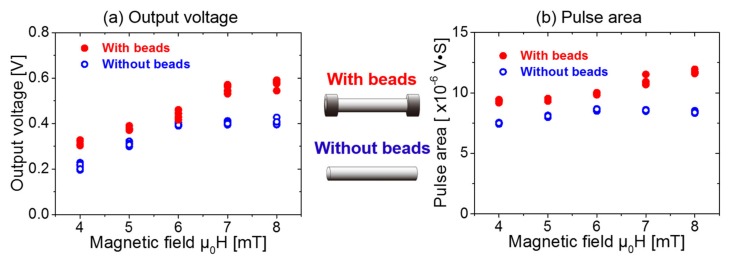
Comparison of the experimental results of the amplitude and the area of the Wiegand pulse with or without ferrite beads, displaying: (**a**) output voltage and (**b**) pulse area. (The meanings of amplitude and area of the pulse are described in Figure 3).

It can be observed from Figure 8 that the amplitude and the area of the Wiegand pulse from the Wiegand sensor increases with an increase in the applied magnetic field. This is irrespective of whether or not the ferrite beads are installed at both ends of the Wiegand wire. The reason for this result is that the volume of the reversed soft layer increases against the hard core based on the increase in the external excitation magnetic field. When all of the soft layer and the hard core are antiparallel, the amplitude and the area of the pulse will reach a maximum. This will also not increase with an increase in the external magnetic field. In summary, the increase in the amplitude and the area of the pulse is attributed to the increase in the volume of the reversed soft layer against the hard core.

However, the amplitude and area of the Wiegand pulse are larger when using ferrite beads than that without ferrite beads. This suggests that the ferrite beads at both ends of the wire are capable of increasing magnetic flux density through the center of the Wiegand wire. Therefore, the volume of the magnetically reversed soft layer is increased, and the amplitude and area of the pulse increase.

Thus, the experimental results show that the addition of ferrite beads at both ends of the Wiegand wire, without changing the external magnetic field, increases the volume of the reversed soft layer against that of the hard core. The amplitude and area of the Wiegand pulse increase, improving the energy of the Wiegand pulse.

### 4.2. Relationship between the Position of the Pickup Coil and Energy

**Figure 9:**
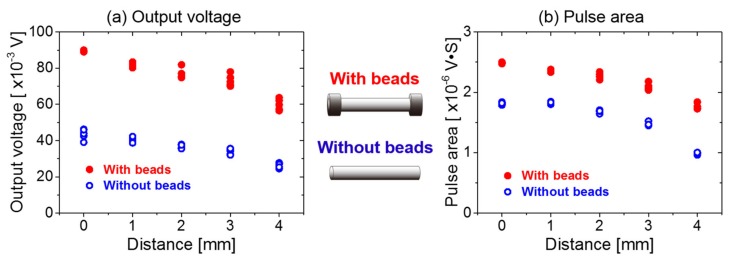
Experimental results of the relationship between the amplitude and area of the Wiegand pulse and the position of the pickup coil, displaying: (**a**) output voltage and (**b**) pulse area.

As shown in Figure 9, as the distance between the pickup coil and the Wiegand wire center increases, the amplitude and the area of the Wiegand pulse decrease. In other words, the energy of the Wiegand pulse decreases. To analyze the causes of this phenomenon, the magnetic field distributions were simulated at a distance of 5 mm from the surface of the magnet applied in the study, as demonstrated in Figure 7a.

It can be seen from Figure 7a that the magnetic field is strong in the middle and weak at both ends. This makes the volume of the reversed soft layer against the hard core larger at the center of the Wiegand wire and smaller at both ends. Therefore, the amplitude and the area of the pulse detected by the pickup coil is the largest at the center of the Wiegand wire. This shows a decreasing trend towards both ends. When the pickup coil is at the center of the Wiegand wire, the detected energy of the Wiegand pulse is at its maximum.

It can also be demonstrated from Figure 9 that, irrespective of where the pickup coil is placed around the Wiegand wire, the amplitude and the area of the pulse output are larger when using the ferrite beads. This further verifies that the setting of the ferrite beads at both ends of the Wiegand wire can improve the volume of the reversed soft layer against the hard core; thus, improving the energy of the Wiegand pulse.

### 4.3. Relationship between the Angle and Energy

**Figure 10:**
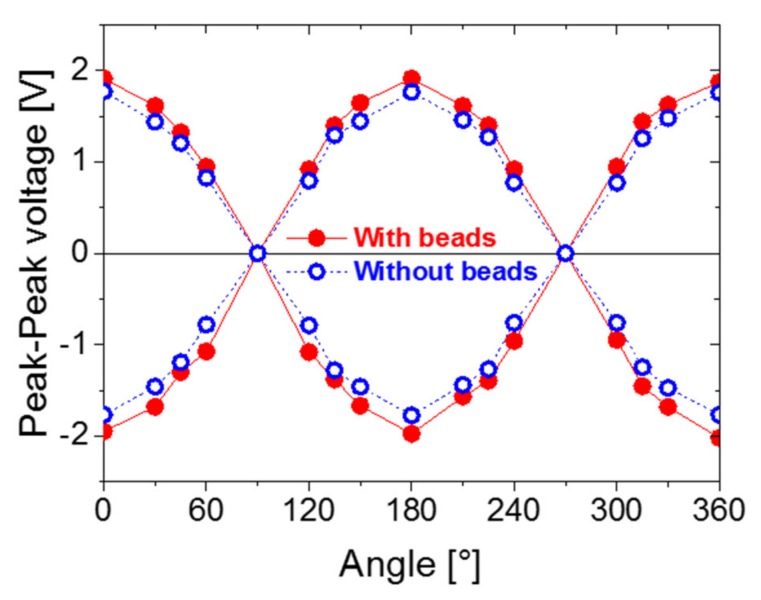
Experimental results of the relationship between the angle of the Wiegand sensor in the applied magnetic field and peak-to-peak voltage of the Wiegand pulse from the Wiegand sensor.

Figure 10 displays the positive and negative peak-to-peak voltages of the Wiegand sensor, from 0° to 360° for both the “With beads” and “Without beads” models. The abscissa in Figure 10 represents the angle between the Wiegand sensor and the magnetic induction line. The ordinate represents the peak-to-peak voltages of the Wiegand pulse for the corresponding angle.

As demonstrated from Figure 10, at the angles of 0°, 180°, or 360° between the Wiegand sensor and the magnetic induction line (in the parallel or antiparallel states), the peak-to-peak value of the Wiegand pulse is at its maximum. In other words, the output energy is at its maximum. On the contrary, when the angle becomes 90° or 270° (in a vertical state), the peak-to-peak value of the Wiegand pulse is at a minimum; thus, the energy output is at its minimum.

In other words, the magnetic induction lines that are parallel or antiparallel can excite the Wiegand sensor successfully, whereas those in the vertical state cannot achieve this. The Wiegand sensor and the magnetic induction line are not in a parallel or antiparallel state and they are inclined at a certain angle. As a result, the magnetic induction lines that excite the Wiegand sensor to output the Wiegand pulses are the parallel or antiparallel components of the magnetic induction lines, which have a particular angle to the Wiegand sensor.

Furthermore, the intensity of the effective magnetic field applied to the Wiegand wire is reduced. That is, the magnetic field, which reverses the soft layer, is reduced, and the volume of the reversed soft layer becomes reduced. As a result, the peak-to-peak voltage of the Wiegand pulse is decreased. However, when the direction of the applied magnetic field is parallel or antiparallel to the Wiegand wire, the peak-to-peak voltage of the Wiegand pulse reaches its maximum. The experimental results shown in Figure 9 support this analysis. Simultaneously, both the positive and negative peak-to-peak voltages of the Wiegand sensor in the “With beads” model are larger than those in the “Without beads” model. Therefore, setting the ferrite beads is advantageous for the practical applications of the Wiegand sensor for the power supply.

## 5. Conclusions

In this study, the energy of the Wiegand pulse was described. Through a large number of experiments, the factors affecting the energy of the Wiegand pulse were determined, and their relationship with the energy of the Wiegand pulse was successfully stated. The essential relationship between these factors and the energy of the Wiegand pulse was determined to have an effect on the volume of the reversed soft layer against the hard core. For the same Wiegand wire, the same pickup coil, and the same external excitation conditions, a larger volume of the reversed soft layer against the hard core could produce a Wiegand pulse with more energy.

When using the Wiegand sensor in practical applications, ferrite beads should be set at both ends of the Wiegand wire, to increase the magnetic flux density. The pickup coil on the Wiegand wire should be distributed evenly on both sides with the center as the origin. In addition, when the Wiegand sensor is used as a power supply, it should be ensured that the magnetic induction line and the Wiegand sensor are parallel or antiparallel, to obtain the maximum energy.
